# General Amyloid Inhibitors? A Critical Examination of the Inhibition of IAPP Amyloid Formation by Inositol Stereoisomers

**DOI:** 10.1371/journal.pone.0104023

**Published:** 2014-09-26

**Authors:** Hui Wang, Daniel P. Raleigh

**Affiliations:** 1 Department of Chemistry, Stony Brook University, Stony Brook, New York, United States of America; 2 Graduate Program in Biochemistry and Structural Biology, Graduate Program in Biophysics, Stony Brook University, Stony Brook, New York, United States of America; Weizmann Institute of Science, Israel

## Abstract

Islet amyloid polypeptide (IAPP or amylin) forms amyloid deposits in the islets of Langerhans; a process that is believed to contribute to the progression of type 2 diabetes and to the failure of islet transplants. An emerging theme in amyloid research is the hypothesis that the toxic species produced during amyloid formation by different polypeptides share common features and exert their effects by common mechanisms. If correct, this suggests that inhibitors of amyloid formation by one polypeptide might be effective against other amyloidogenic sequences. IAPP and Aβ, the peptide responsible for amyloid formation in Alzheimer's disease, are particularly interesting in this regard as they are both natively unfolded in their monomeric states and share some common characteristics. Comparatively little effort has been expended on the design of IAPP amyloid inhibitors, thus it is natural to inquire if Aβ inhibitors are effective against IAPP, especially since no IAPP inhibitors have been clinically approved. A range of compounds inhibit Aβ amyloid formation, including various stereoisomers of inositol. Myo-, scyllo-, and epi-inositol have been shown to induce conformational changes in Aβ and prevent Aβ amyloid fibril formation by stabilizing non-fibrillar β-sheet structures. We investigate the ability of inositol stereoisomers to inhibit amyloid formation by IAPP. The compounds do not induce a conformational change in IAPP and are ineffective inhibitors of IAPP amyloid formation, although some do lead to modest apparent changes in IAPP amyloid fibril morphology. Thus not all classes of Aβ inhibitors are effective against IAPP. This work provides a basis of comparison to work on polyphenol based inhibitors of IAPP amyloid formation and helps provide clues as to the features which render them effective. The study also helps provide information for further efforts in rational inhibitor design.

## Introduction

**Figure 1:**
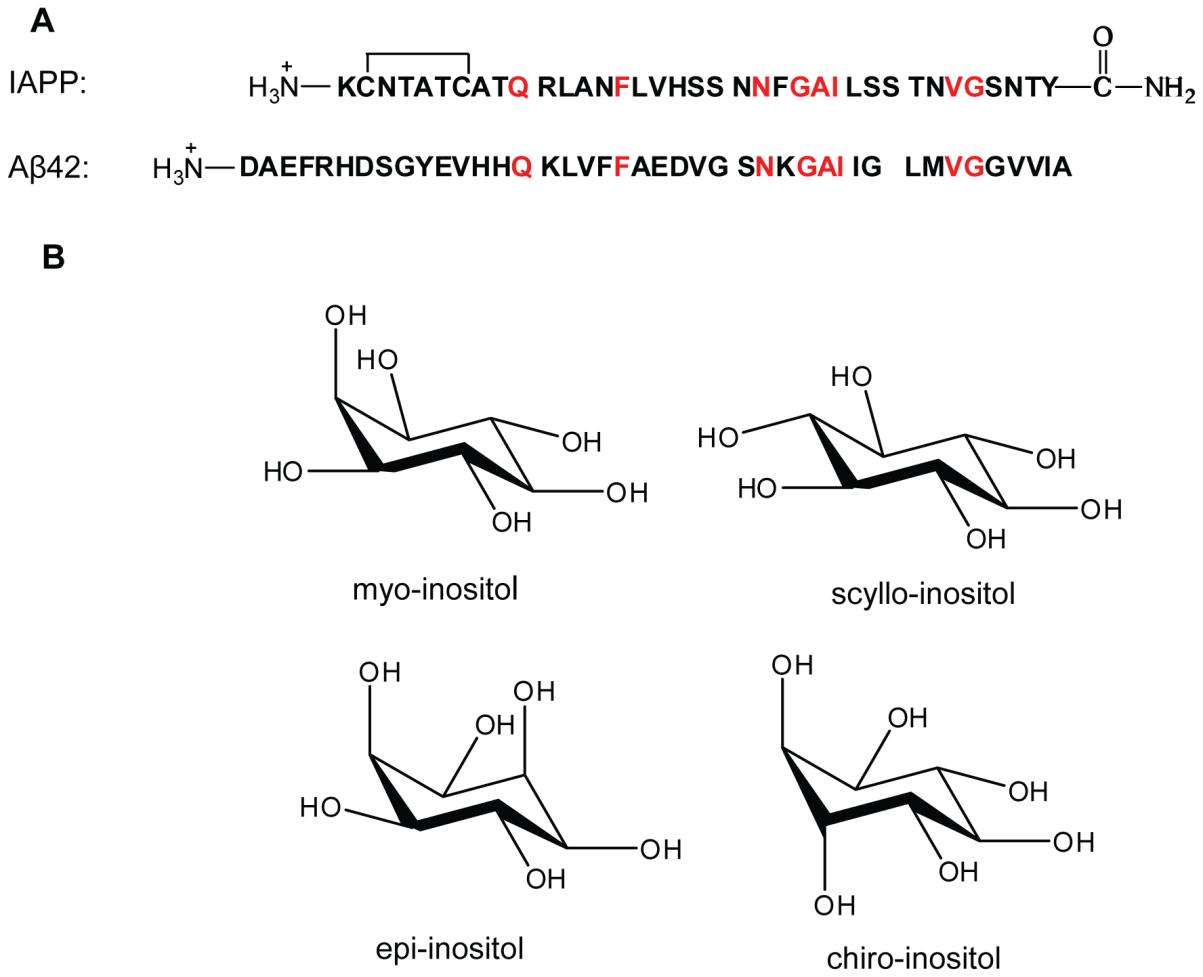
Peptide sequences and inositol structures. (A) Sequence of IAPP and Aβ42. The molecules are aligned according to reference 20. Residues which are identical in both sequences are labeled in red. IAPP has a disulfide bond between Cys 2 and Cys 7 and an amidated C-terminus. (B) Structures of the inositol stereoisomers examined in this work.

Amyloid formation plays a role in a broad range of human diseases including Alzheimer's disease, Parkinson's disease and type 2 diabetes (T2D) [1], [2]. Islet amyloid polypeptide (IAPP or amylin) is a neuroendocrine hormone that forms amyloid deposits in the pancreatic islets of Langerhans in T2D [3], [4]. The peptide normally suppresses postprandial glucagon secretion, helps regulate gastric emptying, and induces satiety, thereby complementing the effects of insulin in glycemic control, but IAPP forms islet amyloid in T2D by an unknown mechanism [5]–[8]. Islet amyloid formation is associated with the reduction of β cell mass in T2D and is believed to contribute to the progression of the disease [9]–[11]. Recent investigations have revealed that islet amyloid contributes to graft failure after islet transplantation [12], [13]. IAPP is produced as a prohormone, and is processed in the Golgi and in the insulin secretory granule, where it is stored. Mature IAPP is 37 residues in length with a disulfide bond between Cys-2 and Cys-7 and an amidated C-terminus (Figure 1). The peptide aggregates aggressively *in vitro* and is toxic to cultured pancreatic islet β cells and islets [14].

An emerging theme in amyloid research is the hypothesis that the toxic species produced during amyloid formation share common physio-chemical features and exert their deleterious effects by common modes [15]–[19]. If correct, this suggests that inhibitors of amyloid formation by one polypeptide might be effective against other amyloidogenic sequences. IAPP and Aβ are particularly interesting in this regard as both are natively unfolded in their monomeric states, and although they have modest sequence identity, they do share some common characteristics (Figure 1) [20]. The two polypeptides are similar in length, 37 versus 40 or 42 residues, are both “natively unfolded”, and both contain conserved aromatic residues. Comparatively little effort has been expended on the design and development of islet amyloid inhibitors compared to that invested in studies of Aβ inhibitors. Thus, it is natural to inquire if Aβ inhibitors are effective against IAPP in light of the features shared by the two polypeptides, and given that amyloid formation by IAPP can be seeded by Aβ [20]. None of the existing IAPP inhibitors have been clinically approved and the mechanism of their action is not well understood. Thus, new classes of IAPP amyloid inhibitors are required [21]–[28].

Several peptide inhibitors, based on the full sequence of IAPP, have been derived that are effective against Aβ *in vitro* [22], [25], [29]. The polyphenol epigallocatechin-3-gallate (EGCG) inhibits amyloid formation by a wide range of natively unfolded polypeptides including Aβ and IAPP, but its mode of action is not clear, and in some cases might involve chemical modification of the target peptide [27], [30]–[32]. Another polyphenol, resveratrol, has also been reported to inhibit *in vitro* amyloid formation by IAPP and Aβ [33], [34]. However, neither polyphenols nor the peptide based inhibitors are “drug like” and it is not clear if the results with these compounds are generalizable.

A range of compounds, in addition to polyphenols, have been reported to inhibit Aβ amyloid formation, including various stereoisomers of inositol, but have not been tested against IAPP. Inositols are a class of cyclohexylpolyols with eight naturally occurring stereoisomers, the physiologically active four of which are: myo-, chiro-, epi- and scyllo-inositol (Figure 1) [35]. Both myo- and scyllo-inositol are found in the human central nervous system, and myo-inositol, the most abundant stereoisomer, is the head group of phosphotidylinositol and plays a role in a range of physiological processes [35], [36]. *In vitro* studies have shown that inositol stereoisomers inhibit amyloid formation by Aβ42 in a stereochemistry-dependent manner. Myo-, scyllo-, and epi-inositol induce conformational changes in Aβ, and prevent Aβ amyloid fibril formation by stabilizing non-fibrillar, β-sheet structures, and have been shown to protect cultured neuronal cells from Aβ-induced toxicity, while chiro-inositol does not have these effects [37], [38]. These studies were conducted with the inositol stereoisomers in large excess, at a peptide to inositol ratio of 1 to 20 by weight (∼1 to 500 by molar). A more recent study of the interaction of Aβ42 and scyllo-inositol used thioflavin-T fluorescence assays to show that the compound, when added in a 10-fold molar excess, only slightly slowed the rate of Aβ42 amyloid formation, but did not abolish fibril formation [39]. The two studies argue that the capability of inositol to inhibit Aβ42 amyloid formation is dose dependent. This conclusion is supported by a recent ion mobility spectrometry study conducted at a peptide to scyllo-inositol ratio of 1 to 1, which revealed that scyllo-inositol reduced the extent of oligomer formation by a fragment of Aβ, Aβ(25–35), but did not prevent the formation of oligomers with β-sheet fibrillar structure [40]. Collectively, the *in vitro* data indicate that scyllo-inositol inhibits Aβ amyloid formation in a dose dependent manner and protects cultured neurons. Scyllo-inositol has also been reported to prevent and reverse the Alzheimer phenotype in a mouse model [41].

This class of compounds has not been investigated as IAPP inhibitors and the most effective small molecule anti-IAPP compounds *in vitro*, polyphenols and sulfated triphenyl methyl compounds, are undesirable as drug leads. Thus, it is important to expand the chemical diversity of potential IAPP inhibitors. Given the similarities between IAPP and Aβ, and the fact that a number of compounds have been shown to be active against both molecules, it is reasonable to examine the effects of different classes of Aβ inhibitors on IAPP. Here we investigate the activity of four inositol stereoisomers to inhibit amyloid formation by IAPP. The compounds do not induce a conformational change in soluble IAPP and are ineffective inhibitors of IAPP amyloid formation, although some do lead to modest changes in IAPP amyloid fibril morphology. Thus not all classes of Aβ inhibitors are effective against IAPP.

The study is also of interest as a basis of comparison to work on polyphenol based inhibitors of IAPP amyloid formation. EGCG, resveratrol, and other polyphenols inhibit IAPP amyloid formation, but the mechanism is not fully understood [26], [27], [33], [42]. EGCG is believed to function by diverting IAPP into non-productive, “off-pathway” aggregates that are not competent to go on to form amyloid [30], [31], [42]. However, the molecular structures of these aggregates are not known. It is not known if the effects are due to their multiple hydroxyl functionalities, their polyphenolic character, their ability, in some cases, to covalently modify proteins, or other features [26]–[28], [32], [42]. The inositols are not aromatic, but are rich in hydroxyl groups that can potentially hydrogen-bond to IAPP, particularly as IAPP has a large number of residues with side chains that contain hydrogen-bond donors and acceptors (Figure 1). The results help provide clues about the features important in polyol and polyphenol based IAPP amyloid inhibitors and may help provide a basis for further rational inhibitor design.

## Materials and Methods

### Peptide Synthesis

IAPP was synthesized using a CEM microwave peptide synthesizer on a 0.1 mmol scale, utilizing 9-fluorenylmethyloxycarbonyl (Fmoc) chemistry. 5-(4′-Fmoc-aminomethyl-3′5-dimethoxyphenol) valeric acid (PAL-PEG) resin was used in order to afford an amidated C-terminus. To improve the yield, Fmoc-protected pseudoproline (oxazolidine) dipeptide derivatives were incorporated as previously described [43]. Standard Fmoc reaction cycles were used as previously described [44]. The first residue attached to the resin, all β-branched residues and all pseudoproline dipeptide derivatives were double-coupled. Standard trifluoroacetic acid (TFA) methods were employed to cleave the peptides from the resin.

### Peptide Oxidation and Purification

After cleavage, crude peptides were dissolved into 20% (v/v) acetic acid and then lyophilized. This step was repeated several times before oxidation to improve the solubility of the peptides. The dry peptides were dissolved into 100% dimethyl sulfoxide at room temperature to promote disulfide formation and were purified via reverse-phase high-performance liquid chromatography using a Vydac C18 preparative column [45]. The purity of the peptide was checked by analytical HPLC before each experiment, and the mass of the peptide was identified by ionization time-of-flight mass spectrometry; IAPP, expected 3903.6, observed 3904.6.

### Sample Preparation

Myo-, scyllo-, and chiro-inositol were purchased from Sigma. Epi-inositol was purchased from TCI America. They were used without further purification. Compounds were dissolved in 20 mM Tris-HCl buffer at pH 7.4 to make 50 mM stock solutions before each experiment. IAPP was dissolved in 100% hexafluoroisopropanol (HFIP) to make a 1.6 mM stock solution. Stock solutions of the peptide were filtered using 0.45 µM Acrodisc syringe filter with a GHP membrane and the required amount of peptide was lyophilized overnight to remove HFIP. Dry peptide was dissolved into Tris-HCl buffer for the fluorescence experiments.

### Thioflavin-T Detected Fluorescence Assays

Amyloid formation was monitored by thioflavin-T binding assays conducted without stirring at 25°C. A Beckman Coulter DTX 880 plate reader with a multimode detector was used to measure the fluorescence, using an excitation wavelength of 430 nm and an emission wavelength of 485 nm. Immediately before the measurement, dry peptide was dissolved into Tris-HCl buffer and thioflavin-T solution, followed by the addition of inositol from stock solutions when inositol was used. The final concentrations were 16 µM IAPP and 32 µM thioflavin-T in 20 mM Tris-HCl (pH 7.4) When inositol was present, the IAPP to inositol ratio was at 1 to 20 by weight. Experiments were repeated three times and reported time constants are average ± standard deviation.

### Circular Dichroism (CD)

Far-UV CD experiments were performed on an Applied Photophysics Chirascan CD spectrophotometer at 25°C. Aliquots from the kinetic experiments were removed at the time points of interest and the spectra were recorded as the average of three repeats over a range of 190–260 nm, at 1 nm intervals. A 0.1 cm quartz cuvette was used and a background spectrum was subtracted from each measurement.

### Transmission Electron Microscopy (TEM)

TEM images were collected at the Life Science Microscopy Center at Stony Brook University. 15 µL aliquots were removed from the solution, blotted on a carbon-coated 200-mesh copper grid for 1 min and then negatively stained with saturated uranyl acetate for 1 min.

## Results and Discussion

### Inositols do not induce a conformational change in IAPP, unlike their effect on Aβ

**Figure 2:**
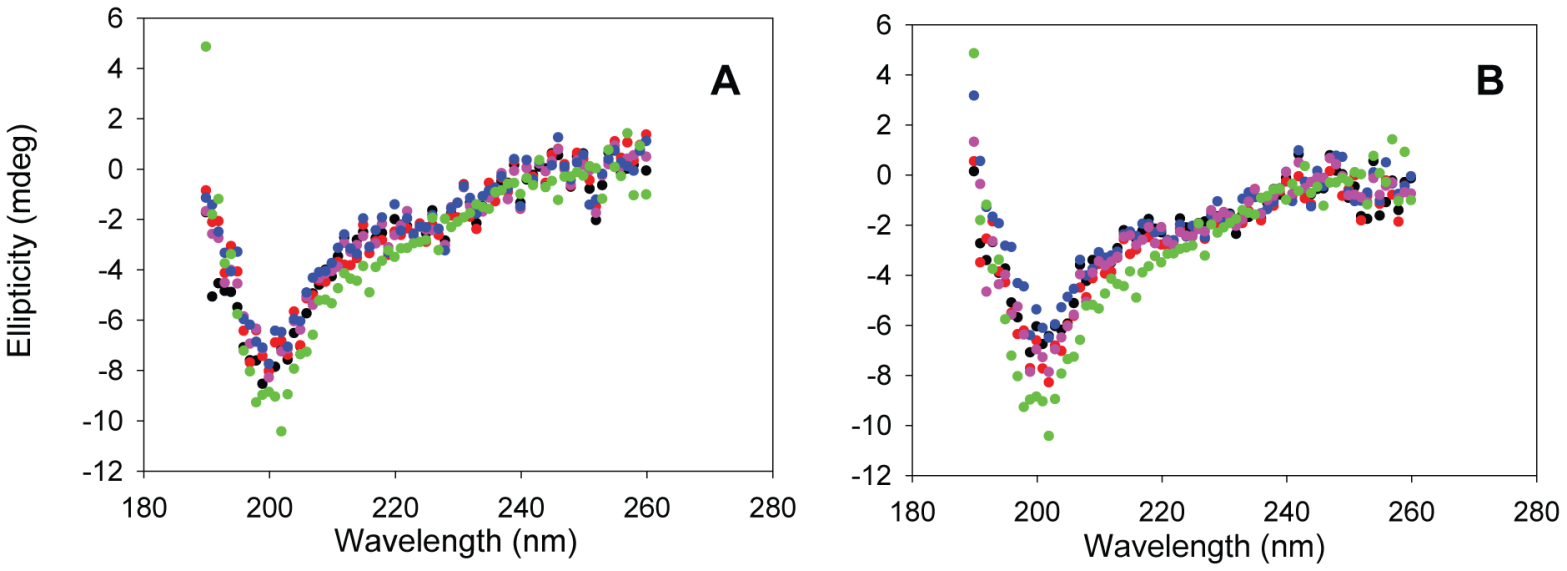
Inositols do not induce a conformational change in IAPP. CD spectra of IAPP in the presence of different inositol stereoisomers at the beginning of the amyloid formation reaction (A) and after 300 min incubation (B). Black, IAPP; red, IAPP + myo-inositol; green, IAPP + epi-inositol; purple, IAPP + scyllo-inositol; blue, IAPP + chiro-inositol. The concentration of IAPP was 16 µM. The IAPP to inositol ratio was 1 to 20 by weight in each mixture. The experiments were conducted in 20 mM Tris-HCl (pH 7.4), without stirring at 25°C.

**Figure 3:**
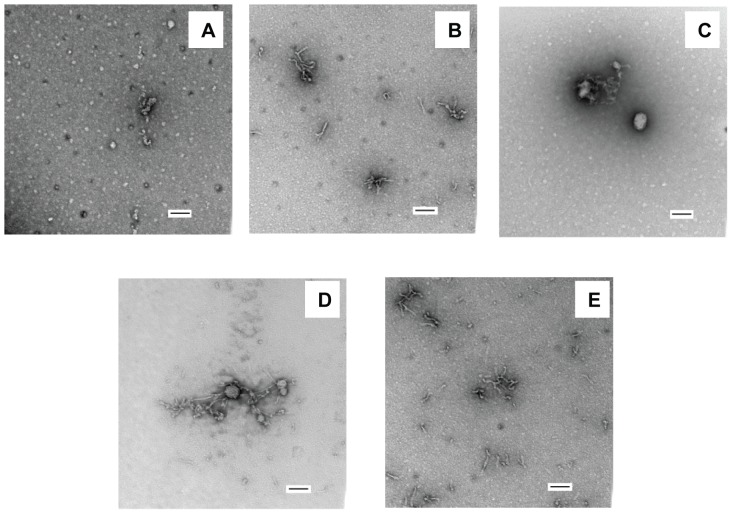
TEM images at the beginning of the amyloid formation reaction. (A) IAPP (B) IAPP + myo-inositol (C) IAPP + epi-inositol (D) IAPP + scyllo-inositol (E) IAPP + chiro-inositol. Scale bars represent 100 nm. The concentration of IAPP was 16 µM. The IAPP to inositol ratio was 1 to 20 by weight in each mixture. The experiments were conducted in 20 mM Tris-HCl (pH 7.4), without stirring at 25°C.

Previous studies have shown that myo-, epi- and scyllo-inositol rapidly induce Aβ42 to form β-structure, while chiro-inositol does not [37], [38]. To test if inositol exerts similar effects on IAPP, we incubated the polypeptide with each stereoisomer separately at a peptide to inositol ratio of 1 to 20 by weight, chosen to mimic the conditions used for the Aβ studies. CD was used to monitor the conformation of IAPP. Immediately after mixing, all of the samples showed typical random coil structure CD spectra which were indistinguishable from the spectrum of IAPP alone (Figure 2), indicating that the compounds exert different effects on IAPP compared to Aβ. TEM images of all of the mixtures were similar to images recorded of freshly dissolved IAPP alone and revealed only a few amorphous aggregates (Figure 3). Under the conditions of our experiments, IAPP forms amyloid with a lag time of about 10 hours. We collected the CD spectra again after 5 hours incubation, a time chosen to be in the middle of the lag phase, but did not observe any obvious conformational change induced by any of the four inositol stereoisomers (Figure 2).

### Inositol is a modest inhibitor of IAPP aggregation, but does not abolish amyloid fibril formation

**Figure 4:**
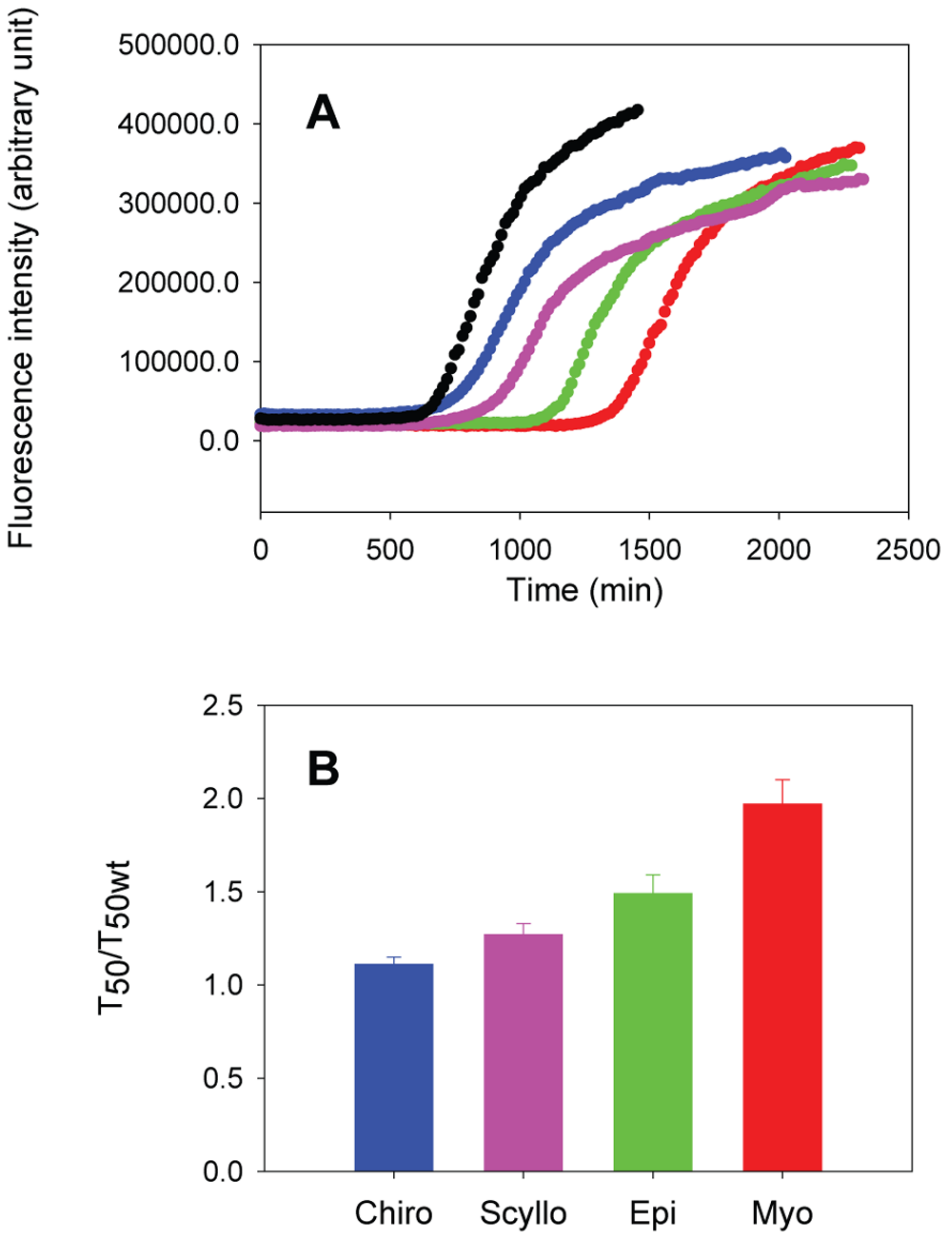
The effect of inositols on IAPP amyloid formation depends on their stereochemistry. (A) Kinetic curves of amyloid formation by IAPP and mixtures of IAPP with inositol stereoisomers as monitored by thioflavin-T fluorescence assays. Black, IAPP; red, IAPP + myo-inositol; green, IAPP + epi-inositol; purple, IAPP + scyllo-inositol; blue, IAPP + chiro-inositol. (B) A bar graph comparing the effects of the different inositols on the value of T_50_. Data is plotted as T_50_ normalized by the T_50_ of IAPP in the absence of any compounds. Error bars represent standard deviations for three repeats. The concentration of IAPP was 16 µM. The IAPP to inositol ratio was 1 to 20 by weight in each mixture. The experiments were conducted in 20 mM Tris-HCl (pH 7.4), without stirring at 25°C.

**Figure 5:**
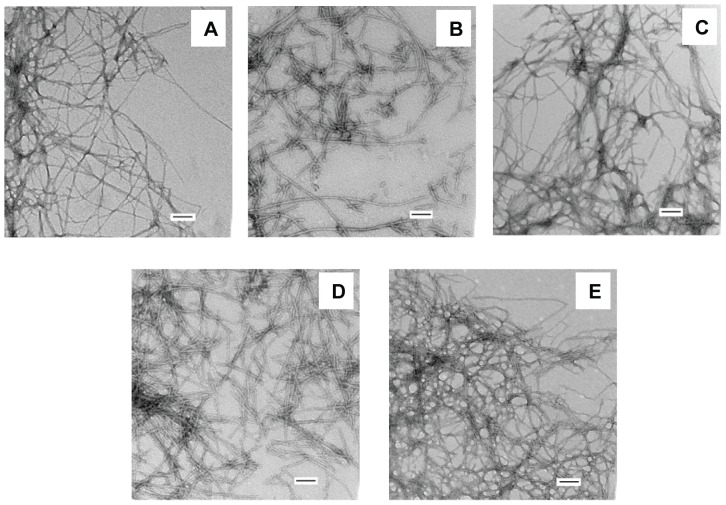
Inositols do not prevent IAPP amyloid formation. TEM images of (A) IAPP, (B) IAPP + myo-inositol, (C) IAPP + epi-inositol, (D) IAPP + scyllo-inositol and (E)IAPP + chiro-inositol were recorded at the end of the amyloid formation reaction. Scale bars represent 100 nm. An aliquot was removed at the end of each kinetic experiment for TEM analysis. The concentration of hIAPP was 16 µM. The IAPP to inositol ratio was 1 to 20 by weight in each mixture. The experiments were conducted in 20 mM Tris-HCl (pH 7.4), without stirring at 25°C.

In the case of Aβ42, three of the isomers proved to be effective at inhibiting amyloid formation. No fibrils were detected during the time course of the experiments conducted in the presence of myo-, epi-, and scyllo-inositol, but fibrils with typical Aβ42 amyloid fibril morphology were observed in the presence of chiro-inositol. We measured the kinetics of amyloid formation by IAPP in the presence of each inositol stereoisomer using fluorescence detected thioflavin-T binding assays. Thioflavin-T is a small dye that undergoes an increase in its quantum yield upon binding to amyloid fibrils and is widely used to monitor the kinetics of amyloid formation. Prior work has shown that the dye does not perturb the kinetics of IAPP amyloid formation under the conditions used here. As shown in Figure 4, the four stereoisomers have modest inhibitory effects on IAPP amyloid formation. Myo-inositol, the most potent inhibitor among the four against IAPP was still only weakly effective, and increased the lag phase of IAPP amyloid formation by a factor of 2.0±0.13, compared to a factor of 1.5±0.10 for epi-inositol and 1.3±0.06 for scyllo-inositol. These are small effects, but they are reproducible. Comparison to the published data on inositol Aβ interactions shows that the compounds are far less effective at inhibiting IAPP amyloid formation [37], [38]. The fourth stereoisomer, chiro-inositol, barely changed the lag time of IAPP in our studies with a T_50_ only 10% longer than in the absence of the compound. The result with chiro-inositol is generally consistent with the results of the earlier work on Aβ. We collected TEM images at the end of each reaction to examine the effects of the different inositol stereoisomers on fibril morphology. The fibrils formed by IAPP in the presence of chiro- or epi-inositol had a morphology that was indistinguishable from that observed for fibrils formed by IAPP alone. In contrast, in the presence of myo- or scyllo-inositol, shorter fibrils were observed (Figure 5). There is no obvious direct correlation between the effects on the lag phase and the effects of the compounds on fibril morphology. This is not surprising given that only small effects are observed on both.

### The potential molecular basis for the different effects of inositols on Aβ and IAPP

The mechanism of inositol-Aβ42 interaction is not fully understood. Molecular dynamics (MD) simulations have led to the hypothesis that scyllo- and chiro-inositol exhibit similar weak binding to a seven residue core fragment of Aβ, Aβ (16–22), which contains a di-phenylalanine sequence. This work suggested that a stereospecific face-to-face stacking mode of scyllo-inositol with the Phe side chains in Aβ plays a role in the ability of the compound to inhibit amyloid formation. The stacking is postulated to result from the interaction of the two hydrophobic planar surfaces in the compound with Aβ. The equatorial position of the hydroxyls in scyllo-inositol leads to two hydrophobic faces, and this, combined with a higher propensity for hydrogen bonding, was proposed to contribute to the observed stereochemistry-dependent activity [46], [47]. The simulations also suggested that inositol inhibits Aβ amyloid formation by preventing the lateral association or stacking of protofibrillar β-sheet oligomers [47]. IAPP contains three aromatic residues, F15, F23 and Y37, but lacks a di-aromatic sequence. The prior results, together with a sequence comparison of IAPP and Aβ, suggest that inositol peptide interactions are sequence specific and may depend on the aromatic sequence in Aβ. An alternative explanation, that we cannot rule out, is that the aggregation pathways of the two peptides might be different and inositols may more effectively target the Aβ pathway.

## Conclusions

We have characterized the ability of myo-, scyllo-, epi- and chiro-inositol to inhibit amyloid formation by IAPP. Their efficacy is stereo-chemically dependent. Chiro-inositol does not affect IAPP amyloid formation, consistent with prior studies on Aβ. However, the remaining three inositol stereoisomers clearly have much less effect upon IAPP amyloid formation than they do on Aβ amyloid formation. Myo-inositol, the most potent inhibitor among the four under our experimental conditions, still had only very modest effects and increased the lag time of IAPP amyloid formation by just 2 fold. In sharp contrast, according to published reports, myo-, epi-, and scyllo-inositol totally abolish amyloid fibril formation by Aβ when added at the same concentration used here [37], [38]. Moreover, these inositol stereoisomers stabilized non-fibrillar β-structure in Aβ, an effect that was proposed to contribute to the inhibition of fibril formation [37], [38]. In contrast, none of the stereoisomers tested were capable of inducing an initial conformational change in IAPP, similar to that seen for Aβ. These observations clearly indicate that the design of amyloid inhibitors is likely to be specific for the protein of interest, and show that, while some compounds can inhibit multiple amyloidogenic proteins, not all inhibitors of one protein will be effective against a similar amyloidogenic peptide.

Myo- and scyllo-inositol did have a modest effect on IAPP fibril morphology and shorter fibrils were observed in their presence. The effects of the inositol stereoisomers on fibril morphology are small, but are dependent on the spatial distribution of the hydroxyl groups. IAPP contains a large fraction of residues with hydrogen bonding functionalities in their side chains (Figure 1), including ten Thr or Ser residues and seven Asn or Gln residues, and these provide multiple potential sites for hydrogen bonding interactions with the inositols.

Certain polyphenols have been proven to be potent *in vitro* inhibitors of amyloid formation by IAPP and Aβ. EGCG is particularly effective and abolishes IAPP amyloid formation when added at a 1 to 1 molar ratio [26]. Resveratrol is less effective, but does inhibit amyloid formation by both peptides. It is not clear if the effects are due to the multiple hydroxyl groups of EGCG, its polyphenolic character, or its susceptibility to spontaneous modifications [32]. The fact that the inhibitory ability of inositol stereoisomers are stereochemistry-dependent, and are much less effective on IAPP amyloid formation than EGCG or resveratrol, indicates that factors other than simply multiple hydroxyl groups are important for polyphenol IAPP interactions [26], [27]. These likely include the aromatic character of the polyphenols, the larger hydrophobic surfaces presented by EGCG, and the distribution of the hydroxyl groups. Along these lines, the polyhydroxyl groups on the planar aromatic rings of EGCG have a very different spatial arrangement than those in the inositols and prior work has suggested that the distribution of the hydroxyls in polyphenols influences their ability to inhibit IAPP amyloid formation [27], [28]. In the case of EGCG, its ability to form covalent linkages with polypeptides may also contribute. The sequence targets of resveratrol and EGCG are not known, although EGCG has been proposed to interact with the peptide backbone and to make relatively non-specific interactions with sidechains [27], [31]. This may account, in part, for the lack of specificity of EGCG. Studies with IAPP analogs have revealed that aromatic residues are not important for IAPP EGCG interactions, while the computational studies summarized above have suggested that di-aromatic sequences could be important for inositol peptide interactions. This too might contribute to the different specificity of EGCG versus inositols.

## References

[1] Sipe JD (1994) Amyloidosis. Crit Rev Clin Lab Sci 31: 325–354. 7888076 10.3109/10408369409084679

[2] Vendruscolo M, Zurdo J, MacPhee CE, Dobson CM (2003) Protein folding and misfolding: a paradigm of self-assembly and regulation in complex biological systems. Philos Trans A Math Phys Eng Sci 361: 1205–1222. 12816607 10.1098/rsta.2003.1194

[3] Cooper GJ, Willis AC, Clark A, Turner RC, Sim RB, et al (1987) Purification and characterization of a peptide from amyloid-rich pancreases of type 2 diabetic patients. Proc Natl Acad Sci 84: 8628–8632. 3317417 10.1073/pnas.84.23.8628 PMC299599

[4] Westermark P, Wernstedt C, O′Brien TD, Hayden DW, Johnson KH (1987) Islet amyloid in type 2 human diabetes mellitus and adult diabetic cats contains a novel putative polypeptide hormone. Am J Pathol 127: 414–417. 3296768 PMC1899776

[5] Gedulin BR, Rink TJ, Young AA (1997) Dose-response for glucagonostatic effect of amylin in rats. Metabolism 46: 67–70. 9005972 10.1016/s0026-0495(97)90170-0

[6] Rushing PA, Hagan MM, Seeley RJ, Lutz TA, D′Alessio DA, et al (2001) Inhibition of central amylin signaling increases food intake and body adiposity in rats. Endocrinology 142: 5035–5038. 11606473 10.1210/endo.142.11.8593

[7] Clementi G, Caruso A, Cutuli VMC, deBernardis E, Prato A, et al (1996) Amylin given by central or peripheral routes decreases gastric emptying and intestinal transit in the rat. Experientia 52: 677–679. 8698109 10.1007/BF01925572

[8] Scherbaum WA (1998) The role of amylin in the physiology of glycemic control. Exp Clin Endocr Diab 106: 97–102. 10.1055/s-0029-1211958 9628238

[9] Clark A, Wells CA, Buley ID, Cruickshank JK, Vanhegan RI, et al (1988) Islet amyloid, increased A-cells, reduced B-cells and exocrine fibrosis: quantitative changes in the pancreas in type 2 diabetes. Diabetes Res Clin Pract 9: 151–159. 3073901

[10] Westermark P, Grimelius L (1973) The pancreatic islet cells in insular amyloidosis in human diabetic and non-diabetic adults. Acta Pathologica Microbiologica Scandinavica Section A Pathology 81A: 291–300. 10.1111/j.1699-0463.1973.tb03538.x 4129056

[11] Westermark P, Wilander E (1978) The influence of amyloid deposits on the islet volume in maturity onset diabetes mellitus. Diabetologia 15: 417–421. 367856 10.1007/BF01219652

[12] Andersson A, Bohman S, Borg LA, Paulsson JF, Schultz SW, et al (2008) Amyloid deposition in transplanted human pancreatic islets: a conceivable cause of their long-term failure. Exp Diabetes Res 2008: 562985. 19277203 10.1155/2008/562985 PMC2652583

[13] Potter KJ, Abedini A, Marek P, Klimek AM, Butterworth S, et al (2010) Islet amyloid deposition limits the viability of human islet grafts but not porcine islet grafts. Proc Natl Acad Sci USA 107: 4305–4310. 20160085 10.1073/pnas.0909024107 PMC2840144

[14] Lorenzo A, Razzaboni B, Weir GC, Yankner BA (1994) Pancreatic islet cell toxicity of amylin associated with type-2 diabetes mellitus. Nature 368: 756–760. 8152488 10.1038/368756a0

[15] Bucciantini M, Giannoni E, Chiti F, Baroni F, Formigli L, et al (2002) Inherent toxicity of aggregates implies a common mechanism for protein misfolding diseases. Nature 416: 507–511. 11932737 10.1038/416507a

[16] Lambert MP, Barlow AK, Chromy BA, Edwards C, Freed R, et al (1998) Diffusible, nonfibrillar ligands derived from A beta(1–42) are potent central nervous system neurotoxins. Proc Natl Acad Sci USA 95: 6448–6453. 9600986 10.1073/pnas.95.11.6448 PMC27787

[17] Bucciantini M, Calloni G, Chiti F, Formigli L, Nosi D, et al (2004) Prefibrillar amyloid protein aggregates share common features of cytotoxicity. J Biol Chem 279: 31374–31382. 15133040 10.1074/jbc.M400348200

[18] Sciacca Michele FM, Kotler Samuel A, Brender Jeffrey R, Chen J, Lee D-k, et al (2012) Two-step mechanism of membrane disruption by Aβ through membrane fragmentation and pore formation. Biophys J 103: 702–710. 22947931 10.1016/j.bpj.2012.06.045 PMC3443794

[19] Kayed R, Head E, Thompson JL, McIntire TM, Milton SC, et al (2003) Common structure of soluble amyloid oligomers implies common mechanism of pathogenesis. Science 300: 486–489. 12702875 10.1126/science.1079469

[20] O′Nuallain B, Williams AD, Westermark P, Wetzel R (2004) Seeding specificity in amyloid growth induced by heterologous fibrils. J Biol Chem 279: 17490–17499. 14752113 10.1074/jbc.M311300200

[21] Yan LM, Tatarek-Nossol M, Velkova A, Kazantzis A, Kapurniotu A (2006) Design of a mimic of nonamyloidogenic and bioactive human islet amyloid polypeptide (IAPP) as nanomolar affinity inhibitor of IAPP cytotoxic fibrillogenesis. Proc Natl Acad Sci USA 103: 2046–2051. 16467158 10.1073/pnas.0507471103 PMC1413694

[22] Abedini A, Meng FL, Raleigh DP (2007) A single-point mutation converts the highly amyloidogenic human islet amyloid polypeptide into a potent fibrillization inhibitor. J Am Chem Soc 129: 11300–11301. 17722920 10.1021/ja072157y

[23] Porat Y, Mazor Y, Efrat S, Gazit E (2004) Inhibition of islet amyloid polypeptide fibril formation: a potential role for heteroaromatic interactions. Biochemistry 43: 14454–14462. 15533050 10.1021/bi048582a

[24] Mishra R, Bulic B, Sellin D, Jha S, Waldmann H, et al (2008) Small-molecule inhibitors of islet amyloid polypeptide fibril formation. Angew Chem Int Ed 47: 4679–4682. 10.1002/anie.200705372 18470855

[25] Meng FL, Raleigh DP, Abedini A (2010) Combination of kinetically selected inhibitors in trans leads to highly effective inhibition of amyloid formation. J Am Chem Soc 132: 14340–14342. 20873820 10.1021/ja1046186 PMC3199963

[26] Meng F, Abedini A, Plesner A, Verchere CB, Raleigh DP (2010) The flavanol (-)-epigallocatechin 3-gallate inhibits amyloid formation by islet amyloid polypeptide, disaggregates amyloid fibrils, and protects cultured cells against IAPP-induced toxicity. Biochemistry 49: 8127–8133. 20707388 10.1021/bi100939a PMC3199968

[27] Cao P, Raleigh DP (2012) Analysis of the inhibition and remodeling of islet amyloid polypeptide amyloid fibers by flavanols. Biochemistry 51: 2670–2683. 22409724 10.1021/bi2015162 PMC3329122

[28] Noor H, Cao P, Raleigh DP (2012) Morin hydrate inhibits amyloid formation by islet amyloid polypeptide and disaggregates amyloid fibers. Protein Sci 21: 373–382. 22238175 10.1002/pro.2023 PMC3375438

[29] Yan LM, Velkova A, Tatarek-Nossol M, Rammes G, Sibaev A, et al (2013) Selectively N-methylated soluble IAPP mimics as potent IAPP receptor agonists and nanomolar inhibitors of cytotoxic self-assembly of both IAPP and Aβ40. Angew Chem Int Ed 52: 10378–10383. 10.1002/anie.201302840 23956012

[30] Bieschke J, Russ J, Friedrich RP, Ehrnhoefer DE, Wobst H, et al (2010) EGCG remodels mature α-synuclein and amyloid-β fibrils and reduces cellular toxicity. Proc Natl Acad Sci USA 107: 7710–7715. 20385841 10.1073/pnas.0910723107 PMC2867908

[31] Ehrnhoefer DE, Bieschke J, Boeddrich A, Herbst M, Masino L, et al (2008) EGCG redirects amyloidogenic polypeptides into unstructured, off-pathway oligomers. Nat Struct Mol Biol 15: 558–566. 18511942 10.1038/nsmb.1437

[32] Palhano FL, Lee J, Grimster NP, Kelly JW (2013) Toward the molecular mechanism(s) by which EGCG treatment remodels mature amyloid fibrils. J Am Chem Soc 135: 7503–7510. 23611538 10.1021/ja3115696 PMC3674815

[33] Mishra R, Sellin D, Radovan D, Gohlke A, Winter R (2009) Inhibiting islet amyloid polypeptide fibril formation by the red wine compound resveratrol. ChemBioChem 10: 445–449. 19165839 10.1002/cbic.200800762

[34] Ladiwala ARA, Lin JC, Bale SS, Marcelino-Cruz AM, Bhattacharya M, et al (2010) Resveratrol selectively remodels soluble oligomers and fibrils of amyloid Aβ into off-pathway conformers. J Biol Chem 285: 24228–24237. 20511235 10.1074/jbc.M110.133108 PMC2911349

[35] Fisher SK, Novak JE, Agranoff BW (2002) Inositol and higher inositol phosphates in neural tissues: homeostasis, metabolism and functional significance. J Neurochem 82: 736–754. 12358779 10.1046/j.1471-4159.2002.01041.x

[36] Michaelis T, Helms G, Merboldt KD, Hanicke W, Bruhn H, et al (1993) Identification of scyllo-inositol in proton NMR-spectra of human brain *in vivo* . NMR Biomed 6: 105–109. 8384468 10.1002/nbm.1940060116

[37] McLaurin J, Franklin T, Chakrabartty A, Fraser PE (1998) Phosphatidylinositol and inositol involvement in Alzheimer amyloid-beta fibril growth and arrest. J Mol Biol 278: 183–194. 9571042 10.1006/jmbi.1998.1677

[38] McLaurin J, Golomb R, Jurewicz A, Antel JP, Fraser PE (2000) Inositol stereoisomers stabilize an oligomeric aggregate of Alzheimer amyloid β peptide and inhibit Aβ-induced toxicity. J Biol Chem 275: 18495–18502. 10764800 10.1074/jbc.M906994199

[39] Sinha S, Du Z, Maiti P, Klärner F-G, Schrader T, et al (2012) Comparison of three amyloid assembly inhibitors: the sugar scyllo-inositol, the polyphenol epigallocatechin gallate, and the molecular tweezer CLR01. Acs Chem Neurosci 3: 451–458. 22860214 10.1021/cn200133x PMC3386858

[40] Bleiholder C, Do TD, Wu C, Economou NJ, Bernstein SS, et al (2013) Ion mobility spectrometry reveals the mechanism of amyloid formation of A beta(25–35) and its modulation by inhibitors at the molecular level: epigallocatechin gallate and scyllo-inositol. J Am Chem Soc 135: 16926–16937. 24131107 10.1021/ja406197f

[41] McLaurin J, Kierstead ME, Brown ME, Hawkes CA, Lambermon MHL, et al (2006) Cyclohexanehexol inhibitors of A beta aggregation prevent and reverse Alzheimer phenotype in a mouse model. Nat Med 12: 801–808. 16767098 10.1038/nm1423

[42] Young LM, Cao P, Raleigh DP, Ashcroft AE, Radford SE (2014) Ion mobility spectrometry-mass spectrometry defines the oligomeric intermediates in amylin amyloid formation and the mode of action of inhibitors. J Am Chem Soc 136: 660–670. 24372466 10.1021/ja406831n PMC3928500

[43] Abedini A, Raleigh DP (2005) Incorporation of pseudoproline derivatives allows the facile synthesis of human IAPP, a highly amyloidogenic and aggregation-prone polypeptide. Org Lett 7: 693–696. 15704927 10.1021/ol047480+

[44] Marek P, Woys AM, Sutton K, Zanni MT, Raleigh DP (2010) Efficient microwave-assisted synthesis of human islet amyloid polypeptide designed to facilitate the specific incorporation of labeled amino acids. Org Lett 12: 4848–4851. 20931985 10.1021/ol101981b PMC3052696

[45] Abedini A, Singh G, Raleigh DP (2006) Recovery and purification of highly aggregation-prone disulfide-containing peptides: application to islet amyloid polypeptide. Anal Biochem 351: 181–186. 16406209 10.1016/j.ab.2005.11.029

[46] Li G, Rauscher S, Baud S, Pomes R (2012) Binding of inositol stereoisomers to model amyloidogenic peptides. J Phys Chem B 116: 1111–1119. 22091989 10.1021/jp208567n

[47] Li G, Pomes R (2013) Binding mechanism of inositol stereoisomers to monomers and aggregates of A beta(16–22). J Phys Chem B 117: 6603–6613. 23627280 10.1021/jp311350r

